# Air Contamination in Operating Theatres: The Key Factors That Can Influence It

**DOI:** 10.1155/cjid/8852879

**Published:** 2025-05-11

**Authors:** Lorenzo Dragoni, Davide Amodeo, Gabriele Cevenini, Nicola Nante, Maria Francesca De Marco, Gabriele Messina

**Affiliations:** ^1^Post Graduate School of Public Health, University of Siena, Siena, Italy; ^2^Department of Medical Biotechnologies, University of Siena, Siena, Italy; ^3^Department of Molecular and Developmental Medicine, University of Siena, Siena, Italy; ^4^Hygiene and Epidemiology Unit, Santa Maria Alle Scotte Teaching Hospital, Siena, Italy

**Keywords:** airborne contamination, air contamination, door opening, operating theatres, surgical site infections

## Abstract

**Objectives:** Adequate ventilation and air filtration in the operating theatre are essential measures to prevent surgical site infections, which impact on hospital stay, healthcare costs and increased risk of mortality. The aim of the study is to assess how other factors, such as the number of operators and the opening of doors during surgery, affect microbiological airborne contamination.

**Methods:** The data were extrapolated from 105 reports of operational controls conducted in the operating rooms in Siena's Teaching Hospital, Italy, from 2018 to 2021. The number of colonies incubated at 22°C and 36°C, was related by Spearman correlation analysis to the number of operators in the rooms and the number of air changes. The Mann–Whitney test was used to assess the difference between the mean of colonies detected with doors closed and opened.

**Results:** The number of colonies incubated at 22°C was correlated only with air changes (Spearman *ρ* = −0.441; *p* < 0.001). In contrast, those incubated at 36°C were correlated with air changes (*ρ* = −0.394; *p* < 0.001) and the number of operators (*ρ* = +0.249; *p*=0.011). For colonies incubated at 22°C, the mean difference between opened and closed doors was not statistically significant (*p*=0.575). In contrast, the difference was statistically significant for those incubated at 36°C (*p*=0.013). In terms of airflow, our study showed a statistically significant difference (*p* < 0.001) between laminar and turbulent flow rooms for both colonies.

**Conclusion:** Continuous monitoring of airflows, correlated with door opening and closing and the number of operators, can help predict levels of microbiological air contamination and thus prevent surgical infections.

**Summary**

• What does this study add to existing knowledge?
◦ Our study assesses the key variables of airborne microbiological contamination in operating theatres, focussing on the role of the operators present and their impact on this highly controlled environment in terms of the particles they move and the type of contamination they carry.
• What are the key implications for public health interventions, practice or policy?
◦ Our study could provide a better understanding of how to implement best practice to minimise contamination in theatres and therefore potential surgical site infections.

## 1. Introduction

Operating theatres (OTs) are an essential part of the hospital as they are where surgeries and other medical procedures are performed. To ensure the safety and well-being of patients, these rooms must have consistently low levels of airborne and surface microbial contamination. In fact, the quality of the environment in these rooms is of paramount importance as it directly impacts the patient's outcome and can affect the procedure's success; therefore, control of microbiological contamination is crucial to promote patient safety and minimize the risk of infection. For this reason, routine checks are carried out in OTs both ‘at rest' to validate the efficiency of the systems, and ‘in operational', i.e., to verify whether, during operational phases, parameters such as airborne microbiological contamination and microclimate allow safe and comfortable operations [1, 2].

However, although the controls can verify the requirements of the standard [3], other elements can affect the system's efficiency; these include technological aspects (e.g., laminar/turbulent flows), behavioural aspects, and overcrowding of rooms. Healthcare workers are the primary source of contamination in OTs, and their actions and behaviours can significantly impact the room's cleanliness level [4].

Another factor that can influence the level of contamination in OTs is the presence of equipment and surfaces. This can include surgical instruments, medical devices and other items used in the operating room that need to be properly sterilized or disinfected according to their use [5].

Many studies have investigated the levels of microbiological contamination of OTs by relating them to construction and technological aspects; on the other hand, there is less knowledge of how inappropriate behaviour on the part of operators (ex. Limiting the opening and closing of the OT doors/windows) can compromise the efficiency of the system and how the progressive presence of operators in the room can increase the risk of airborne microbiological contamination even though ventilation systems are in place. For these reasons, the aim of the research is to verify the microbial contamination levels according to: (i) the operators behaviours; (ii) their presence and the (iii) the role of environmental parameters.

## 2. Methods

### 2.1. Setting

This cross-sectional study was conducted in the teaching hospital of Siena, Italy, from January 2018 and September 2021. The University Hospital of Santa Maria alle Scotte has about 600 beds, 3000 employees, as well as students of medicine and health professions, resident doctors and students in training. The hospital covers more than 170 thousand square metres and is divided into 7 buildings. The hospital has 26 OTs, divided into pavilions according to the type of intervention. Four are dedicated to gynaecological and paediatric surgery (located at pavilion 4, floor 3), three to general surgery emergencies and orthopaedic surgery (located at pavilion 1, floor 2), four to general surgery (located at pavilion 1, floor 2), seven to neurosurgery, maxillofacial and cardio thoraco-vascular surgery (located at pavilion 2, floor 2), three to interventional radiology/neuroradiology (located at pavilion 2, floor 0) three to ophthalmological and ENT surgery (located at lot pavilion 1, floor 8) and finally two to interventional cardiology (pavilion 3, floor 3).

The OTs may vary due to the type of air flow (laminar or turbulent), size, arrangement of the doors/windows and the operating table, number of air change per hours. In Table 1 are summarized the main characteristics of the 26 OTs.

**Figure 1:**
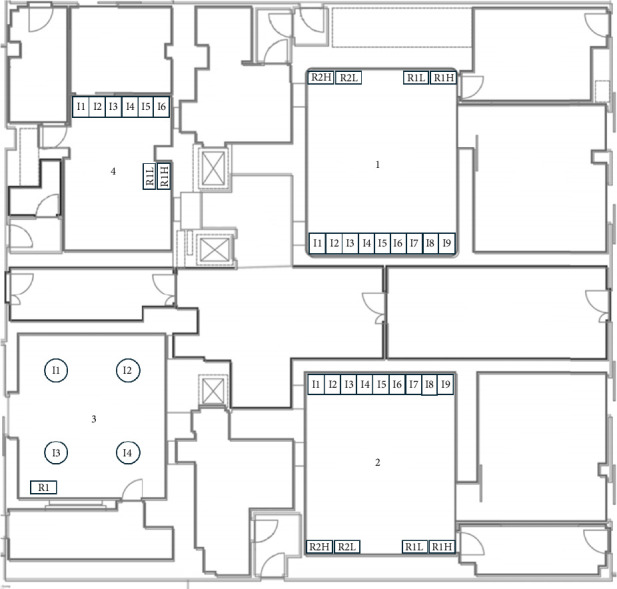
Gynaecological and paediatric rooms layout. 1. Paediatric; 2. Gynaecological; 3. Gynaecological emergency; 4. Ambulatory surgery; *I* = air intake, *R* = air return, *H* = high, *L* = low.

**Figure 2:**
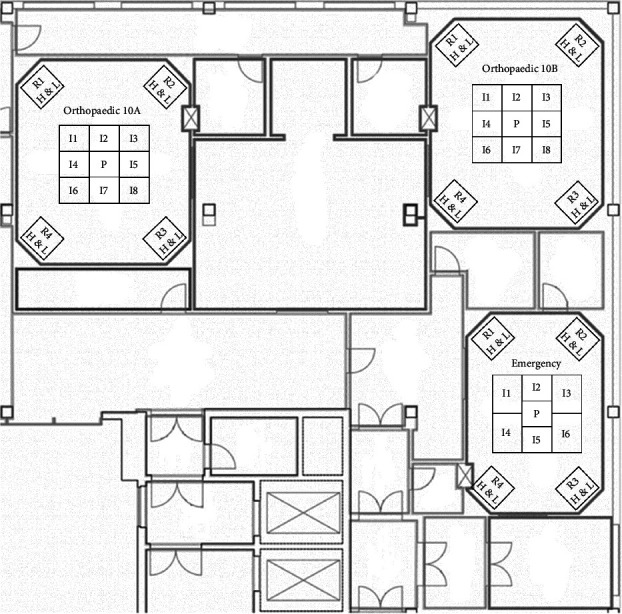
Emergency and orthopaedic rooms layout. *I* = air intake, *R* = air return, H & L = high and low, *P* = plenum.

**Figure 3:**
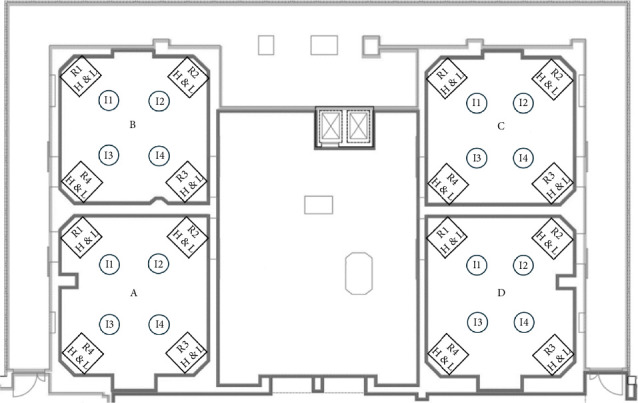
General surgery rooms layout. *I* = air intake, *R* = air return, H & L = high and low.

**Figure 4:**
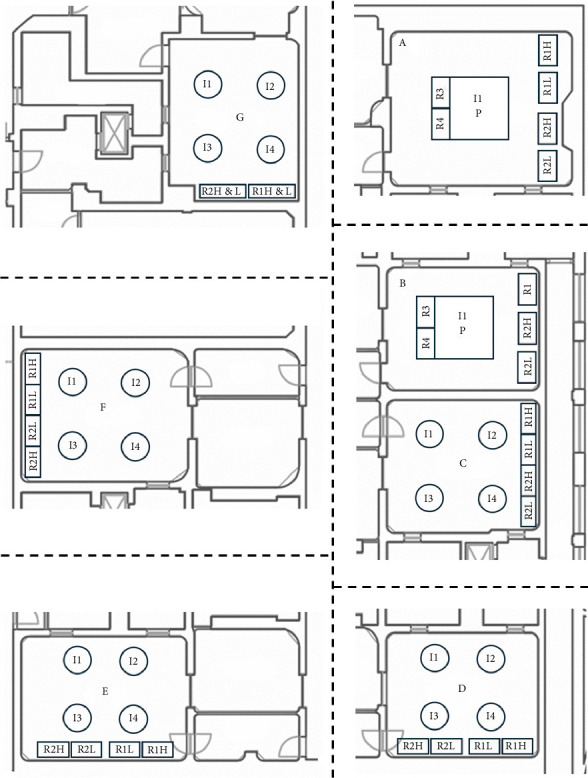
Neurosurgery and cardiac rooms layout. *I* = air intake, *R* = air return, H & L = high and low, *H* = high, *L* = low, P = plenum.

**Figure 5:**
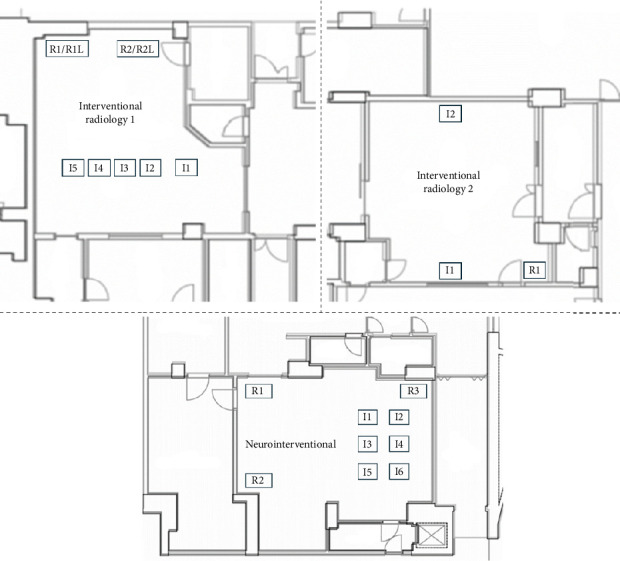
Interventional neuroradiology rooms layout. *I* = air intake, *R* = air return, *L* = low.

**Figure 6:**
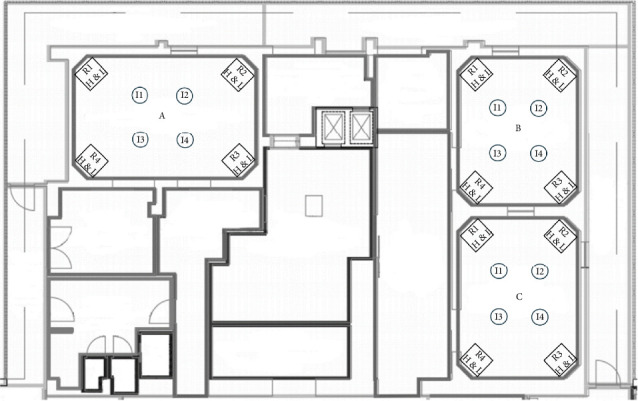
Ophthalmological and ENT rooms layout. *I* = air intake, *R* = air return, H & L = high and low.

**Figure 7:**
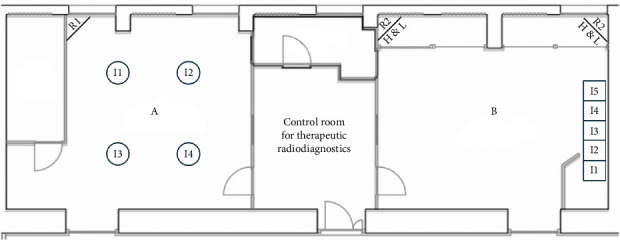
Interventional cardiology rooms layout. *I* = air intake, *R* = air return, H & L = high and low.

More specifically the description of the 26 OTs is the following:

- In the gynaecological (123.1 m^3^) and paediatric (123.5 m^3^) OTs, we have a main door for patient entry, which is adjacent to a room for patient preparation and recovery. There is also a second door in each room, which gives access to a room where staff can wash their hands. In both rooms there are two sash windows that communicate with ancillary rooms. The airflows in both rooms are turbulent, with the air supplies at the top and inclined along one side of the room, and the returns along the whole of the opposite side. The gynaecological emergency room and the ambulatory surgery room both have turbulent flow and two windows. The first one has two doors, one leading to the hand washing room, ceiling air supplies with a single return in the corner; while the second one has two doors leading to the patient preparation room and the hand washing room, air supplies at the top and inclined along one side of the room and has high and low returns in the middle of the wall with the two windows (Figure 1).
- The two orthopaedic theatres (141 m^3^ each) share a common area for pre-op preparation and patient recovery. This area provides access to the two OTs through a door. Each room also has a sash window that communicates with the ancillary rooms. The air flows, laminar, from top to bottom and there are high and low air returns in the four corners of the rooms. At the same level, there is an emergency OTs (105 m^3^), which has a main door for patient access and a secondary door that communicates with a patient monitoring area; there is another doorless access point on the opposite side of the secondary door, which leads to a materials/instrument's storage area. There is also a sash window that communicates with an ancillary room. Airflow is turbulent and is directed from top to bottom through a plenum, with air returns at the top and bottom corners of the room (Figure 2).
- The four general surgery rooms (*A* = 102 m^3^, *B* = 95 m^3^, *C* = 91.5 m^3^, *D* = 103 m^3^) are served by a common area where the operators can wash their hands and have a main door for patient entry and an opposite secondary door for material exit. There are no windows. Turbulent air intake is via four ceiling vents; there are two air returns, one high and one low, for each corner of the OTs (Figure 3).
- The neurocardiac surgery block consists of seven rooms ranging in volume from 110 m^3^ to 164 m^3^. The two cardiac OTs have a pre-room for patient preparation and monitoring, which communicates with the OTs through a main door. In addition to a secondary door for scrub personnel, it has two bayonet windows that communicate with ancillary rooms. Laminar flow is from top to bottom through a plenum, while there are both ceiling and wall return zones. The other five rooms have two doors leading to the preoperative room and the hand-washing room respectively and two windows; in addition, they have ceiling air supplies and high and low returns on one side of the room (Figure 4).
- Interventional radiology has two rooms, both with turbulent airflow and volumes of 136 m^3^ and 109 m^3^, respectively. The bigger one has no windows and three doors connecting it to the technical rooms and to the patient preparation and rest room; it has five air supplies in a line on the ceiling and high and low air returns on one side of the room. Number two has no window and four doors connecting it to the staff washrooms, the control room and the patient preparation and rest room; it has two ceiling supplies outlets on opposite sides of the room and one supply outlet in a corner. In the same block there is also a neurointerventional room with turbulent airflow, no windows and two doors, one leading to the reporting room and the other to the filter area; it has six ceiling supplies arranged in two rows and three air returns in the corners of the room (Figure 5).
- The ophthalmic and ENT surgical block has three OTs (*A* = 93.3 m^3^; *B* = 79.9 m^3^; *C* = 73.1 m^3^). All rooms have one window and a door leading to a handwashing area for the operators; there is also a second door in room A which gives access to the patient preparation room. The airflow is turbulent and there are ceiling air supplies and high and low returns at the four corners of the room (Figure 6).
- The two interventional cardiology rooms have respectively four (A) and three (B) doors and no windows, with one of the doors giving access to the control room for therapeutic radio-diagnostics. Room A has a volume of 109 m^3^ and has a turbulent air flow with supply air at the ceiling and a single inlet in the top left corner, while room B has a volume of 140 m^3^, turbulent air flow, supply air at the right side of the ceiling and high and low intakes in the two top corners of the room (Figure 7).

All reports of the controls carried out between January 2018 and September 2021 were analysed after data extraction. In case the number of operators was not recorded the OT was not included in the analysis. The final number of inspections analysed was 105.

### 2.2. Regulations and Standard

The 2009 ISPESL Guidelines on Occupational Safety and Hygiene Standards in the Surgical Ward provide the limits to which we have referred, and they are based on specific ISO technical standards. The three objectives of the ISPESL Guidelines are:

1. To ensure and verify the parameters and hygienic/structural requirements necessary for the proper functioning of the operational theatres;
2. To adopt effective compensatory procedures where necessary;
3. Carry out any preventive and corrective actions necessary to achieve appropriate quality standards.

In compliance with the regulations in force in Italy (DPR *n*. 37 of 14 January 1997 and Legislative Decree 81/08) and taking into account the main technical reference for the microclimate inside OTs (UNI EN ISO 7730: 2006), they establish the following parameters: [3] internal temperature in winter and summer between 20°C and 24°C; relative humidity in summer and winter between 40% and 60%; 15–20 outdoor air changes per hour (without recirculation). The noise level is set at LPS 48 dBA for unidirectional flow and LPS 45 dBA for turbulent flow. In addition, the standard UNI EN ISO 7726: 2002 defines the minimum characteristics of instruments for measuring physical quantities in an environment and specifies methods for measuring physical quantities in this environment.

Furthermore, the UNI 11425: 2011 standard provides guidelines for the design, installation, commissioning, operation and maintenance, qualification and control of contamination, controlled ventilation and air conditioning systems that contribute to clean air in power plant units. This standard defines that it is appropriate for the OTs to be at least class:

- ISO5 for complex surgical procedures such as transplants, prosthesis implantation, neurosurgery, oncology, orthopaedics, with a duration of more than 60 min.
- ISO7 for surgical procedures without implantation of foreign material such as minor surgery, vascular, obstetrics, ophthalmology.
- ISO8 for short duration procedures such as visceral surgery, day surgery, urology. For particulate matter, the maximum allowable particle concentration for each particle size considered, which is different for each ISO class, is determined by ISO 14644-1: 2015.

In particular, with regard to microbiological contamination of the air, since there are currently no technical regulations in Italy that define precise values, the above guidelines refer to the indications contained in the specific standard of the British National Health Service-Health Technical Memorandum 2025 [6], which establishes the following values for microbiological contamination of the air in the proximity of the operating table: for a conventional OTs at rest, with a turbulent flow system, the value is ≤ 35 CFU/m^3^ and for contamination of the air in a conventional OTs at activity, the values are ≤ 180 CFU/m^3^ with a turbulent flow system and ≤ 20 CFU/m^3^ with a unidirectional flow system.

### 2.3. Types of Measurements During Inspections

Based on regulations and standards, the inspections included the collection of measurements for the following parameters:

i. Hourly air changes (ACH) (m^3^/h) through the measurement of the supply air relative to the room volume measured by balometer Testo 420 (Testo SE and Co. KGaA, Titisee-Neustadt, Germany).
ii. Microclimatic measurements with Delta OHM HD 32.3 microclimatic control unit (Delta OHM—Senseca Italy Srl, Padova, Italy): standard and radiant temperature (°C), relative humidity (%); air velocity (m/s) and thermal comfort with PMV (Met 1.6 Clo 0.8) and PPD (Met 1.6 Clo 0.8). These measurements were carried out at rest and in operational.
iii. Airborne particulate matter measured using Climet CI550 (Climet Instruments Company, Redlands, California, USA) and 2 Lasair III 310C microbiological particle counter (Particle Measuring Systems Inc., Boulder, Colorado, USA), at rest conditions, sampling the air at 7 different points in the room; each point was sampled several times to minimize turbulences during the preparation of the measurements. Each sample, 28.3 L/min, measured particle sizes > 0.3, > 0.5 and > 5 with relative mean, standard deviation, standard error, Upper Control Limit (UCL) and ISO class (compared to the ISO class of the project).
iv. The pressure gradient (Pascal) measured using differential manometer Testo 420; (Testo SE and Co. KGaA, Titisee-Neustadt, Germany), at rest condition, between the inside of the OT (positive) and the adjacent environments (negative).
v. The noise level (dbA), at rest condition, measured using Delta Ohm Phonometer HD2010UC; (Delta OHM-Senseca Italy Srl, Padova, Italy).
vi. The microbiological control of surfaces, after the preparation of the OT and at rest condition, was carried out by sampling various points in the OTs (operating bed, machine, trolley, floor, wall, air vent and operating lamp) using 55 mm diameter contact plates, filled with generic Plate Count Agar medium (PCA) (VWR, Radnor, Pennsylvania, USA) to detect Colony Forming Unit (CFU). The PCA medium is composed of the following formulation 5.00 g/L casein peptone, 2.50 g/L yeast extract, 1.00 g/L dextrose, 14.0 g/L agar. After sampling, the contact plates were incubated at 22°C and 36°C, and CFU count manually read after 48 H.
vii. Microbiological control of the air was carried out according to a previous study [7]. Briefly, 4 SAS Aquaria microflow (Aquaria Srl, Milano, Italy) were used with 55 mm diameter RODAC plates, containing PCA medium (VWR, Radnor, Pennsylvania, USA), to sample 1 m^3^ of the air at rest and operating conditions (for each 1 m^3^ 4 Petri dishes, one per 4 positions of the OT, 250 L/each at 120 L/min). The CFU, present in the plates incubated at 22°C and 36°C, were counted at 24 and 48 h after the samplings.

In addition, in operation condition, we recorded also the number of operators present in the operating room and whether the microbiological air sampling was taken having the OT door open/closed.

### 2.4. Statistical Analysis

The correlation between number of colonies incubated at 22°C and 36°C and the number of operators in the rooms and the number of air changes was tested by Spearman correlation analysis. Student's *t*-test or Mann–Whitney rank test were used, depending on normality or non-normality data distribution respectively, to assess the difference of CFU means between doors closed and opened, and between theatres with laminar flow and those with turbulent flow. Normality was tested using the Shapiro–Wilks test. All inferential statistical analyses, performed with the Jamovi software (Ver. 2.5.6), were evaluated with a significance level of 95% (*p* < 0.05).

## 3. Results

The number of colonies incubated at 22°C was correlated only with air changes (Spearman rho = −0.441; *p* < 0.001). In contrast, those incubated at 36°C were correlated with air changes (rho = −0.394; *p* < 0.001) and the number of operators (rho = +0.249; *p*=0.011) (Table 2).

Table 3 shows the descriptive statistics of colony groups at 22°C and colonies at 36°C in relation to the door's status. The table shows the mean, median, standard deviation and standard error of the two groups. These descriptive statistics show that the mean and median CFU are higher in the samples collected with the doors open than in those collected with the doors closed at both 22° and 36° incubation. Given the non-normal distribution (determined by the Shapiro–Wilk test), we performed our statistical analysis using the nonparametric Mann–Whitney test. For what concern closed and opened doors at 22°C, the differences were not statistically significant (Mann–Whitney test, *p*=0.575); in contrast, the CFU differences were statistically significant for those incubated at 36°C (Mann–Whitney test, *p*=0.013) (Table 4).

Table 5 shows the descriptive statistics of 22°C and 36°C colony groups according to airflow type. As in Table 3, this table shows the mean, median, standard deviation and standard error of the two groups. In this case, regardless of incubation at 22° or 36°, the mean and median values are higher for samples taken in OTs equipped with turbulent flow than in those equipped with laminar flow. Given the non-normal distribution (determined by the Shapiro–Wilk test), we performed our statistical analysis using the nonparametric Mann–Whitney test. Analyzing the turbulent and laminar flow type, the differences were statistically significant (Mann–Whitney test, *p* < 0.001) for both colonies incubated at 22°C and those incubated at 36°C (Table 6).

## 4. Discussion and Conclusions

The systems and monitoring actions within an operating room remain an important aspect of ensuring patient safety during surgery. Among the various parameters examined, only a few were found to influence the level of microbiological air contamination. One of the important parameters for ensuring a low level of airborne microbiological contamination is air changes, as they work by continuously diluting environmental pollutants, thereby reducing the presence of microbes. Additionally, the mode of air supply, whether laminar or turbulent, can be a differentiating factor in microbial contamination. Regarding the type of flow, the study by Erichsen Andersson et al. [8] shows that the laminar system reduces the air contamination rate by 89% compared to DV systems under live conditions, Knudsen et al. [9] found that laminar flow reduced the number of CFUs by both active air sampling and passive bacterial sedimentation load compared to TAF OTs. However, in a recent meta-analysis, Ouyang et al. [10] claim that implementation of the laminar systems does not result in a significant reduction in the incidence of surgical site infections, airborne bacterial counts or the incidence of SSIs in orthopaedic operating rooms.

This comparison might seem contradictory; however, the elements being focussed on are different. While it is hypothesized that a lower microbial presence in the air could lead to a lower incidence of SSIs, it is unclear what threshold should be referenced to assume that this relationship is always correlated. There are, of course, other factors that can influence the microbial presence in the air, such as air fluid dynamics, the number of personnel, and their behaviours. These are aspects we have sought to examine and integrate with what is already known in the literature. The results of our study agree with what Erichsen Andersson et al. and Knudsen et al. pointed out in their work, in fact the difference, both for colonies incubated at 22° and those incubated at 36°, was statistically significant (*p* < 0.001), confirming a lower concentration in OTs with laminar flow. In fact, we recorded an average of 11.3 CFUs incubated at 22° in the turbulent flow rooms compared to an average of only 3.77 in the laminar flow rooms. We observed the same trend for colonies incubated at 36°, which averaged 43.6 DFU in the turbulent and 17.6 CFUs in the laminar (Table 5). In a future study, it would be interesting to track the incidence of surgical site infections in the operations we measured, to compare with the work of Ouyang et al.

In a previous study [11], we analysed the association between air changes and microbiological contamination in the OT and found that contamination decreased with increasing number of air changes, although not linearly; we also found that, according to the ISPSL guidelines, 15 ACH is sufficient to stay below the limit of 180 CFUs in theatres with turbulent ventilation, but not in theatres with laminar flow ventilation, which should be below 20 CFUs. Again, in this study, we observed that even when you go below 15 CFUs, you remain in a state where you do not exceed the 180 CFUs limit. This finding allows for an important consideration, namely the existence of other factors that may influence the level of contamination.

The effect of the open/closed status of doors on contamination is also controversial. In their study, Perez et al. [12] observed a relationship between door openness and contamination levels that only existed outside the LAF, and Montagna et al. [13] also claimed to have found no difference in microbial counts when doors were open or closed. In contrast, the studies by Smith et al. [14] and Birgand et al. [15] found this relationship to exist, with the former showing an increase in bacterial counts of around 70% each time a door was opened. Our study agrees with the latter two studies cited above, indeed, for colonies incubated at 36°C, our work showed a statistically significant difference between the two means (*p* < 0.040). Colonies incubated at 22°C had an average of 8.35 CFU with the doors closed and 10.40 CFU with the doors open, while those incubated at 36° had an average of 27.3 CFU–46.1 CFU (Table 3). In our study, we did not consider the number of door openings and closings as in the above studies, but only the state of the doors and windows during surgery now of sampling. We can therefore only confirm the correlation between the state of the doors and the number of CFUs, without being able to give a precise weighting to each door opening event, as Smith et al. do in their paper (each door opening increases the number of contaminated plates by almost 70%).

Similarly, our study shows that colonies incubated at 36° are also correlated with the number of operators in the operating room. This result is consistent with the study by Perez et al. [12], whereas the number of operators (divided into ranges) increases, the number of CFU in the OT increases in a statistically significant manner as in our study (rho = +0.249; *p*=0.011). In contrast, the results of the work by Montagna et al. [13] do not reveal a statistically significant association between the number of operators and increased CFU counts. Studies by Birgand et al. [15] and Cao et al. [16] suggest that the level of activity and movement of operators is more important than the absolute number of operators. Certainly, limiting both the number of operators present and their movements is the most desirable intervention to reduce contamination levels due to personnel.

A comparison between our study and the literature cited does not provide a clear picture of the factors that have the greatest impact on air contamination in the OT, particularly in relation to door opening and the absolute number of operators present. An important difference is that our work does not look at just a few variables but considers all those involved in the contamination of the OTs.

Our work may be limited by the fact that there are fewer laminar than turbulent flow OTs. There is also a lack of some data on the condition of the doors, and in some cases the number of operators has been simulated. However, in addition to the large number of samples, one of the advantages of our work is the high degree of variability in the parameters of the study. This is due to the environment of the OTs analysed, which is never the same. In fact, there are rooms with one door and one window, and others with no windows but three or four doors. There are also different types of air supply, for example with turbulent flow from above, or with turbulent flow but with an oblique direction from one side of the room to the other. The resulting large number of combinations makes the interpretation of the results more heterogeneous.

Considering the results of our study, we can conclude that reducing the number of operators and limiting the opening of operating room doors as much as possible is important to minimize the microbiological load in the air and, consequently, the potential risk of surgical site infections. Healthcare facilities should adopt policies and practices that limit access to the operating room to essential personnel, in addition to ensuring a controlled and well-ventilated environment. This approach will help to improve surgical outcomes and patient safety. Additionally, in our sampling, we measured all the variables necessary for proper control of OTs, such as temperature, relative humidity, operator thermal comfort and differential pressure. At first glance, these variables do not appear to have as significant an impact on contamination levels as the number of operators and the condition of the doors and were therefore not included in the variables to be described in our study. Further studies may reveal new relationships, for example between door condition, differential pressure and contamination levels.

## Figures and Tables

**Table 1:** Operating theatres design features.

Department	Room	Pre-room	Room volume (m^3^)	Hourly changes	Air intake	Air return	Flow type	Flow direction	Doors	Windows
Interventional cardiology	A	No	109	5.6	4	1	Turbulent	Oblique	4	0
Interventional cardiology	B	No	140	22.8	5	4	Turbulent	Oblique	3	0
Gynaecological and paediatric surgery	Paediatric A	Yes	123.5	16.6	9	4	Turbulent	Oblique	2	2
Gynaecological and paediatric surgery	Gynaecological B	Yes	123.1	16	9	4	Turbulent	Oblique	2	2
Gynaecological and paediatric surgery	Ambulatory surgery	No	73	8.67	6	2	Turbulent	Oblique	2	2
Gynaecological and paediatric surgery	Gynaecological emergency	Yes	105.9	10.3	4	1	Turbulent	Top to bottom	2	2
Emergency acceptance dep.	Orthopaedic surgery 10B	Yes	141	41.4	8/Plenum	8	Laminar	Top to bottom	1	1
Emergency acceptance dep.	Orthopaedic surgery 10A	Yes	141	36.2	8/Plenum	8	Laminar	Top to bottom	1	1
Emergency acceptance dep.	Emergency	Yes	105	18	6/Plenum	8	Turbulent	Top to bottom	2	1
General surgery	A	No	102	15.6	4	8	Turbulent	Top to bottom	2	0
General surgery	B	No	95	16.33	4	8	Turbulent	Top to bottom	2	0
General surgery	C	No	91.5	18.4	4	8	Turbulent	Top to bottom	2	0
General surgery	D	No	103	18.6	4	8	Turbulent	Top to bottom	2	0
Neurocardiac surgery	A	Yes	164.4	32	1/Plenum	5	Laminar	Top to bottom	2	2
Neurocardiac surgery	B	Yes	110	60.5	1/Plenum	5	Laminar	Top to bottom	2	2
Neurocardiac surgery	C	Yes	126	7.8	4	4	Turbulent	Top to bottom	2	2
Neurocardiac surgery	D	Yes	118.2	9.1	4	4	Turbulent	Top to bottom	2	2
Neurocardiac surgery	E	Yes	127.9	10.4	4	4	Turbulent	Top to bottom	2	2
Neurocardiac surgery	F	Yes	137.6	12.2	4	4	Turbulent	Top to bottom	2	2
Neurocardiac surgery	G	Yes	130.9	9.3	4	4	Turbulent	Top to bottom	2	2
Neuroimaging and neurointerventional	Neurointerventional	No	149	6.8	6	3	Turbulent	Top to bottom	2	0
Neuroimaging and neurointerventional	Interventional radiology 2	No	136	13.1	5	4	Turbulent	Oblique	3	0
Neuroimaging and neurointerventional	Interventional radiology 2	No	109	6.6	2	1	Turbulent	Top to bottom	4	0
Ophthalmic and ENT surgery	A	Yes	93.3	13.2	4	8	Turbulent	Top to bottom	2	1
Ophthalmic and ENT surgery	B	No	79.9	10.9	4	8	Turbulent	Top to bottom	1	1
Ophthalmic and ENT surgery	C	No	73.1	11.6	4	8	Turbulent	Top to bottom	1	1

**Table 2:** Correlation matrix between total number of colonies incubated at 22°, at 36°, hourly changes and number of operators present.

		Total CFU 22°	Total CFU 36°	Hourly changes	Operators number
Total CFU 22°	Spearman rho	—			
	df	—			
	*p* value	—			

Total CFU 36°	Spearman rho	0.770⁣^∗∗∗^	—		
	df	102	—		
	*p* value	< 0.001	—		

Hourly changes	Spearman rho	**−0.441 **⁣^∗∗∗^	**−0.394 **⁣^∗∗∗^	—	
	df	103	102	—	
	*p* value	< 0.001	< 0.001	—	

Operators number	Spearman rho	0.109	**0.249 **⁣^∗^	0.366⁣^∗∗∗^	—
	df	101	100	101	—
	*p* value	0.271	0.011	< 0.001	—

*Note:* The values in bold indicate the significant correlations between the variables examined, which are referred to in the text of the article.

⁣^∗^*p* < 0.05.

⁣^∗∗^*p* < 0.01.

⁣^∗∗∗^*p* < 0.001.

**Table 3:** Descriptive statistics of colony groups at 22° and colonies at 36° in relation to the door's status.

Incubation Temperature	Group⁣^∗^	CFU Number	Mean	Median	SD	SE
22°C	0	23	8.35	4.00	11.63	2.425
	1	48	10.40	5.00	12.38	1.787

36°C	0	23	27.30	19.00	29.87	6.229
	1	48	46.08	36.50	37.63	5.432

⁣^∗^0 = Closed doors; 1 = opened doors.

**Table 4:** Results of independent samples *t*-test on the difference in the number of colonies incubated at 22° or 36° between open and closed doors.

Incubation Temperature	Test	Statistics	df	*p*	Mean difference	Difference SE
22°C	Student *t*	−0.6649	69.0	0.508	−2.048	3.080
	**Mann**–**Whitney *U***	**506**		**0.575**	**−1.000**	

36°C	Student *t*	−2.0951	69.0	0.040	−18.779	8.963
	**Mann**–**Whitney *U***	**349**		**0.013**	**−16.000**	

*Note:* Values in bold indicate the most appropriate statistical test, which is referenced in the article text.

**Table 5:** Descriptive statistics of 22° and 36° colony groups according to airflow type.

Incubation Temperature	Group⁣^∗^	CFU Number	Mean	Median	SD	SE
22°C	0	83	11.3	7.00	14.05	1.542
	1	22	3.77	1.00	8.15	1.74

36°C	0	82	43.6	34.00	35.65	3.937
	1	22	17.64	8.50	25.03	5.34

⁣^∗^0 = Turbulent; 1 = laminar.

**Table 6:** Results of the independent samples *t*-test for the difference in the number of colonies incubated at 22° or 36° between operating theatres with turbulent flow and those with laminar flow.

Incubation Temperature	Test	Statistics	df	*p*	Mean difference	Difference SE
22°C	Student *t*	2.42	103	0.017	7.58	3.13
	**Mann**–**Whitney *U***	**402.5**		**< 0.001**	**5.00**	

36°C	Student *t*	3.20	102	0.002	25.92	8.10
	**Mann**–**Whitney *U***	**409.0**		**< 0.001**	**21.00**	

*Note:* Values in bold indicate the most appropriate statistical test, which is referenced in the article text.

## Data Availability

The data that support the findings of this study are available from the corresponding author upon reasonable request.
